# Association of novel markers of liver disease with neonatal liver disease in premature baboons, *Papio sp*.

**DOI:** 10.1371/journal.pone.0228985

**Published:** 2020-03-09

**Authors:** Laura M. Keller, Stephanie Eighmy, Cun Li, Lauryn Winter, Jay Kerecman, Zachary Goodman, Naveen Mittal, Cynthia L. Blanco

**Affiliations:** 1 Department of Neonatology, San Antonio Military Medical Center, San Antonio, TX, United States of America; 2 Department of Pediatrics, Brooke Army Medical Center, San Antonio, TX, United States of America; 3 Texas Biomedical Research Institute, San Antonio, Texas, United States of America; 4 Department of Animal Science, Texas Pregnancy and Life-course Health Research Center, University of Wyoming, Laramie, Wyoming, United States of America; 5 Department of Pediatrics, University of Texas Health Science Center at San Antonio, San Antonio, TX, United States of America; 6 Division of Neonatology, Department of Pediatrics, Eastern Maine Health System, Bangor, ME, United States of America; 7 Department of Pathology, Center for Liver Diseases, Inova Fairfax Hospital, Falls Church, VA, United States of America; 8 Division of Neonatology, Department of Pediatrics, University Health System, San Antonio, TX, United States of America; Texas A&M University, UNITED STATES

## Abstract

Parenteral Nutrition (PN) Associated Liver Disease (PNALD) affects up to 60% of neonates; however, techniques for diagnosing and monitoring disease progression remain limited. The neonatal baboon model may provide a unique opportunity to identify serologic markers associated with this disease. The purpose of this study was to investigate if Hyaluronic Acid (HA), TIMP metallopeptidase inhibitor 1 (TIMP1), Amino-terminal Propeptide of Type-III Collagen (PIIINP) and Enhanced Liver Fibrosis (ELF) score associate with histological liver disease in neonatal baboons exposed to PN. Preterm baboons delivered via c-section at 67% gestation received PN for 14 days with or without Intralipid (PRT+IL, PRT-IL, respectively) or were sacrificed after birth (PRTCTR). Term baboons were sacrificed after birth (TERMCTR) or survived 14 days (TERM+14d). Serum HA, TIMP1, and PIIINP concentrations were measured by ELISA. A blinded pathologist assigned liver histological scores following necropsy. HA increased 9.1-fold, TIMP1 increased 2.2-fold, and ELF score increased 1.4-fold in PRT-IL compared to PRTCTR. ALT, AST, and GGT were within normal limits and did not vary between groups. A trend towards increased fibrosis was found in PRT-IL baboons. Microvesicular hepatocyte steatosis and Kupffer cell hypertrophy were elevated in PRT-IL vs PRTCTR. HA and TIMP1 were significantly elevated in preterm baboons with early histological findings of liver disease evidenced by hepatic steatosis, Kupffer cell hypertrophy and a trend towards fibrosis whereas traditional markers of liver disease remained normal. These novel markers could potentially be utilized for monitoring early hepatic injury in neonates.

## Introduction

Parenteral Nutrition (PN) Associated Liver Disease (PNALD) affects 40–60% of neonates and incidence increases with duration of exposure [1]. PNALD places a significant health burden upon these infants and is associated with hepatic cholestasis, coagulopathy, increased infection, and liver fibrosis. In a small percentage of cases, PNALD rapidly progresses to liver failure that requires transplant or results in death [1]. The most common serologic markers used to track PNALD- direct bilirubin, aspartate aminotransferase (AST), alanine aminotransferase (ALT), and gamma-glutamyl transferase (GGT) lack accuracy and do not predict stage of liver fibrosis or project advancement towards liver failure [2–7]. For this reason, liver biopsy remains the gold standard. However, liver biopsy provides only a snap in time assessment and is subject to significant sample error as well as intra- and inter-observer variation, all of which can lead to misdiagnosis of overall liver disease [8,9]. In addition, the procedure is costly and exposes the infant to serious potential complications.

In adult and pediatric populations, several serum markers are being used both singly and in algorithms to assess liver disease and project level of fibrosis [3,4,10–18]. These markers include glycoproteins (hyaluronic acid (HA), laminin, and YKL-40), members of the collagens family of proteins (propeptide of Type III Collagen (PIIINP) and collagen type IV), as well as collagenases and collagenase inhibitors (matrix metalloproteases (MMP) and TIMP metallopeptidase inhibitor 1 (TIMP1)) [17]. Of these markers, HA has been most extensively studied, demonstrating promise as an early indicator of prognosis in biliary atresia in pediatric populations [12] and as a marker of severe hepatic fibrosis in patients with non-alcoholic fatty liver disease (NAFLD) [16]. Furthermore, elevated TIMP1 has also been shown to correlate with hepatic fibrosis in cystic fibrosis liver disease [13]. These “direct” serum markers, so called because they reflect the deposition and removal of extracellular matrix in the liver, can also be combined with “indirect” markers from routine blood tests (AST, ALT, platelet count, etc) into more sophisticated scores, thereby improving the diagnostic utility of these markers for both adult and pediatric patients [3,10,13]. A number of such scoring algorithms have been developed and are available for commercial use including the Enhanced Liver Fibrosis Test (ELF, iQur Ltd, Southampton, UK) [4,15,17,18]. The ELF score combines TIMP1, PIIINP, and HA serum concentrations and has been demonstrated to predict moderate fibrosis and cirrhosis in adults and children [19].

To our knowledge, none of these markers have yet been investigated for use in neonatal hepatic disease. If they were found to be applicable to neonatal liver disease, they could potentially decrease the need for invasive liver biopsy and improve monitoring of overall disease state. In particular, there are concerns about the dose of intralipid and its role in the development of liver disease [20]. As these novel markers require a minimal amount of blood and are fairly noninvasive, it would be possible to repeat the tests for assessing trends over time; raising the possibility of early identification of the subset of infants moving towards rapid liver failure, allowing interventions to be made before reaching transplant criteria. While it is difficult to obtain liver samples in healthy and sick neonates to correlate with serum levels, another mode of exploration exists. Baboons have a 97% phylogenetic proximity with humans, and if born prematurely, develop conditions unique to preterm infants [21]. Moreover, neonatal baboons have been demonstrated to be a highly translational model for evaluating cholestasis and liver dysfunction in extremely low birth weight infants, with pathologic changes in NICU-treated baboons comparable to findings in human infants [22]. The purpose of this study was to investigate if Hyaluronic Acid (HA), TIMP metallopeptidase inhibitor 1 (TIMP1), and Amino-terminal Propeptide of Type III Collagen (PIIINP) alone or combined as an Enhanced Liver Fibrosis (ELF) score, associate with histological liver disease in neonatal baboons exposed to PN with and without intralipids.

## Methods

### Animal care

Blood serum, fixed liver tissue, and frozen liver tissue were obtained from shared control animals from various ongoing studies, as described below. Animals (*Papio anubis* and *Papio cynocephalus* x *Papio anubis* hybrids) were obtained from the Texas Biomedical Research Institute (TBRI) in San Antonio, Texas and the Oklahoma Primate Center (Fort Reno, OK). Experiments were conducted at the TBRI and at the University of Texas Health Science Center at San Antonio (UTHSCSA, San Antonio, TX). The Institutional Animal Care and Use Committees at the TBRI and UTHSCSA approved all studies. Animal experiments were conducted in accordance with accepted standards of humane animal care, and all efforts were made to minimize suffering. There were no early deaths of preterm or term animals. Prior to euthanasia, preterm animals were anesthetized with Ketamine (5 mg/kg, Putney, Portland, USA) and Midazolam (0.1mg/kg, Akorn, Lake Forest, USA) IV and term animals were anesthetized with Ketamine (10mg/kg; IM) and isoflurane gas (1–2%) and titrated to effect. All animals were euthanized via exsanguination followed by pentobarbital. Necropsies were performed immediately following euthanasia.

### Gestational controls

Six preterm baboons were delivered via scheduled Caesarean Section (C/S) under general anaesthesia at 67% (125 ± 2 days) gestation and euthanized immediately after delivery (PRTCTR). Mothers of preterm animals were returned to the TBRI Primate Center after 2–3 weeks to allow recovery from C-Section (described in greater detail below). A total of nine baboons were delivered at term via spontaneous vaginal delivery and breastfed by their mothers for up to 3 days before transfer to UTHSCSA. Mothers of term animals were returned to their colonies following infant transfer. Six of these infants were euthanized at DOL 3–5 (TERMCTR). The remaining three animals were survived for 14 days, and received no interventions prior to euthanasia (TERM+14d). All term animals were housed in temperature-controlled environments, bottle-fed Similac Advance infant formula (Abbott, Abbott Park, IL) every 3–4 hours and monitored by veterinary staff daily until euthanasia.

### Preterm animals

Premature baboons were delivered via C/S under general anaesthesia at 67% gestation. Mothers of preterm baboons received 2 doses of intramuscular antenatal steroids at 48 and 24 hours prior to C/S. Dosing was equivalent to that given to humans in mg/kg body weight. Immediately following delivery, infants were anesthetized with ketamine (5–10 mg/kg) and Midazolam (0.1mg/kg) IM, intubated and given 4ml/kg surfactant (Beractant, Abbott Laboratories, Abbott Park, IL) and then were chronically ventilated with PN for 14 days. Animals received 24-hour care provided by qualified staff members and were monitored by veterinary staff daily. Animals received Ketamine (5–10 mg/kg) and Midazolam (0.1 mg/kg) IV every 2–6 hours to maintain sedation and analgesia. Ventilator management was adjusted per a detailed protocol according to blood gas analysis. Central venous and arterial lines were placed shortly after birth for fluid management, PN, and lab sampling. One group received Intralipid (IL; Fresenius Kabi, Bad Homburg vor der Höhe, Germany) solution starting at 1gm/kg/day and advanced stepwise daily by 0.5 gm/kg/day to a max 3 gm/kg/day (PRT+IL, n = 6). The other group did not receive any IL (PRT-IL, n = 8). For both the PRT+IL and PRT-IL groups, PN was started within 24 hours of delivery and was similar in content and volume to that used in human neonatal intensive care unit patients. Amino acids were started at 1.75g/kg/day and advanced to a max of 3.5g/kg/day by the 2^nd^ day of life (Trophamine, B. Braun Medical, Bethlehem, PA). Dextrose was started at 5% and adjusted to maintain blood glucoses within goal range according to the individual protocol. Standard neonatal dosed multivitamins and trace elements (M.V.I. Pediatric, Pfizer, New York, NY) were added. Electrolytes and total fluid goal were adjusted based on serum chemistries and fluid status. When stable, animals were allowed trophic enteral orogastric feeds to further mimic human neonatal intensive care unit conditions at about 20ml/kg/day (Similac Special Care, Abbott Nutrition, Columbus, GA). Animals were euthanized at 14 ± 2 days of life. Additional details of their daily management and feedings have been published in detail elsewhere [21,24]. Following the C-Section procedure, mothers of preterm infants were monitored twice daily by veterinary staff until removal of sutures at 10–14 days post-surgery. Mothers received Meloxicam (Putney; first dose: 0.2mg/kg, following doses: 0.1mg/kg, IM once daily) or Buprenex (Abbott Animal Health, Abbott Park, USA; 0.01mg/kg, IM twice daily) for analgesia following surgery. Penicillin (Durvet, Blue Springs, USA; 30,000 IU/kg IM, every other day for six days) was given to prevent infection. Once the surgery site was completely healed and the animals cleared by a veterinarian, mothers were returned to the TBRI Primate Center.

### Additional procedures

As previously described, the euglycemic hyperinsulinemic clamp is a technique to measure insulin sensitivity [23,24]. At the start of each procedure, animals were anesthetized with ketamine (10 mg/kg) and isoflurane gas and two peripherally inserted central catheters were placed. Each clamp procedure lasted 120 minutes and consisted of a bolus dose (150 mU/kg, given over 1 minute) of insulin (Novolin; Novo Nordisk Pharmaceuticals, Princeton, USA) followed by a continuous infusion rate of 15 mU·kg^-1^·min^-1^. The glucose infusion rate was simultaneously adjusted to maintain euglycemia (blood glucose concentration of 68–80 mg/dL). Blood samples were collected every 5–10 minutes to monitor blood glucose levels and at -180, 0, +30, +60, +90, and +120 min for analysis of insulin concentration. Animals remained anesthetized for the duration of the experiment and were monitored constantly for signs of discomfort or adverse reactions (increased or decreased heart rate; hyper- or hypotension, or other vital sign changes). Muscle biopsies are performed during the insulin clamp. Animals are appropriately anesthetized prior to muscle biopsies and local anesthesia utilizing 1% lidocaine was used. Biopsies were taken from the biceps femoris muscle via sharp dissection and utilizing sterile technique to obtain ~10 mg of muscle tissue (approximately 3 small pieces of 2–4 mm each). Six TERMCTR and four PRT-IL animals underwent this procedure prior to euthanasia.

### Blood analyses

Serum was collected prior to euthanasia and stored at -80°C. Serum HA, TIMP1, and PIIINP concentrations were measured by ELISA (Hyaluronan Quantikine ELISA [R&D Systems; Minneapolis, MN], TIMP-1 Quantikine ELISA [R&D Systems], PIIINP ELISA [Cusabio Biotech Co., LTD; Houston, TX]). The ELF score was determined using the formula as per N. Alkhouri, et al [25]:

ELFScore=(−7.412)+(0.681*lnHAng/ml)+(0.775*lnPIINPng/ml)+(0.494*lnTIMP1ng/ml)+10.

Average fasting serum glucose levels were measured and compared in all animals prior to necropsy to ensure no significant impairment in gluconeogenesis or metabolic abnormality was present. Direct Bilirubin, Total Bilirubin, ALT, AST, and GGT were measured using standard techniques for n = 6 animals per group (except for TERM+14d, where n = 3).

### Tissue and morphometric analyses

At necropsy, liver tissue was collected and either snap-frozen in LN_2_ or fixed in 10% normal buffered formalin for 24 hours, then transferred to 70% ethanol, and embedded in paraffin. Slides were cut and Hematoxylin and eosin, Masson trichrome, Perl’s, CD68, and Cytokeratin 7 (CK7) stains were prepared. A single liver pathologist, who was blinded to clinical and laboratory data, performed the histological analysis as follows: Histological scores were assigned per standard METAVIR staging [26,27]. Briefly, fibrosis was scored as 0 = none, 1 = portal fibrosis without septa, 2 = portal fibrosis with few septa, 3 = bridging fibrosis, and 4 = cirrhosis. Additional histologic evaluation of PNALD was performed similarly to a foundational study performed by Kerecman et al [22]. Extramedullary hematopoiesis was scored according to the method of Miranda [28]: hepatic erythroid hematopoiesis was scored from 0–2, with 0 = no areas of hematopoietic foci, 1 = widely spaced hematopoietic foci, and 2 = closely opposed hematopoietic foci. Portal and Lobular Myelopoiesis were scored from 0–3 with 0 = absent myelopoiesis, 1 = isolated myeloid cells, 2 = scattered cell clusters, and 3 = frequent clusters or confluent myeloid cells in portal spaces. Acinar bile ducts were noted to be present or absent and expressed as a percent of portal tracts containing at least one duct, with 20 portal tracts counted per animal. Additional semi-quantifiable histopathological parameters were scored from 0–3 with 0 = none, 1 = mild/few, 2 = moderate, and 3 = many/marked to include Kupffer cell hypertrophy, Kupffer cell hemophagocytosis, micro and macrovesicular hepatocyte steatosis, hepatocyte apoptotic bodies and lobular atrophy, bile stasis and Kupffer and hepatocyte iron storage, as described by Kerecerman et al [22]. The presence of CD68+ macrophages was evaluated in the same manner. Assessment of CK7+ ductular reaction and progenitor cells was evaluated according to the method of Eleazar et al: 0 = rare or no CK7+ progenitor cells or ductular reaction around few to no portal tracts, 1 = rare single or focal CK7+ ductular reaction and/or focal clustering of CK7+ progenitor cells around most portal tracts, 2 = continuous CK7+ ductular reaction and/or clusters of CK7+ progenitor cells around <50% of portal tracts, and 3 = continuous CK7+ ductular reaction and/or clusters of CK7+ progenitor cells around >50% of portal tracts [29]. Ductular reaction was defined as irregular cords of cuboidal to flattened, elongated CK7+ cells with small or inapparent lumens and progenitor cells were defined as small CK7+ cells with scant cytoplasm, oval nuclei, and occasional cytoplasmic extensions [29]. Scores were assigned based on assessment of the full slide.

### RT-PCR analysis

Relative mRNA expression of genes involved in inflammation and fibrosis was measured using RT-PCR. RNA was extracted from approximately 20 mg of frozen tissue using the Trizol (Thermo Fisher Scientific, Waltham, USA) methodology followed by clean up using the RNeasy Mini Kit (Qiagen, Germantown, USA). cDNA was synthesized using the Applied Biosystems High Capacity cDNA transcription kit (Thermo Fisher Scientific) according to the manufacturer’s instructions. Taqman primer/probe sets and mastermix (Thermo Fisher Scientific) were used to measure relative gene expression of: NFKB inhibitor alpha (NFKBIA, assay ID: Hs00153283_m1), Collagen Type 1 alpha 1(COL1A1, assay ID: Hs00164004_m1), Fibronectin 1 (FN1, assay ID: Hs01549976_m1), and Actin Alpha 2, smooth muscle (ACTA2, assay ID: Hs05005341_m1). The BioRad CFX384 Touch Real-Time PCR Detection System (BioRad Laboratories, Hercules, USA) was used for conducting the PCR reactions. Relative quantitation of gene expression was accomplished using the relative standard curve method. The quantity of each gene was normalized to importin 8 (IPO8, assay ID: Hs00914057_m1).

### Statistical analysis

All authors had access to the study data and reviewed and approved the final manuscript. Statistical calculations were performed utilizing SPSS 25.0 and STATA. Using a 2-sided test, an alpha of 0.05 and a beta of 0.8, 5 animals were needed per group to determine statistical significance in HA levels. One-way ANOVA with Bonferroni correction was used for comparison of continuous data between more than 2 groups and p-value of p<0.05 was considered statistically significant. Kruskal-Wallis with post-hoc testing (STATA) was used for analyzing categorical data between groups. The p-value for statistical significance was adjusted to p<0.0025 for Kruskal-Wallis testing. The utility of each serum biomarker for discriminating fibrosis levels and other markers of liver disease was assessed using area under the receiver operating characteristic curve analysis and Spearman’s correlation coefficient was used to assess the correlation between serum biomarkers and markers of liver disease. Results are reported as mean ± SEM, unless otherwise specified.

## Results

All relevant data underlying this manuscript can be found archived at DOI: 10.6084/m9.figshare.8326532.

### Demographics

Demographic characteristics of the study animals, including gestational age, male to female ratio, and birth weight are summarized in Table 1. Birth weights were similar between like gestations (Table 1). Serum glucose was similar between like gestations and within normal limits (Table 1). One baboon was excluded from the PRT+IL group final analysis due to severe illness (related to extreme prematurity) leading to early death (final number analyzed in group n = 5).

**Table 1:** Baseline characteristics of infant baboons.

	PRTCTR	PRT+IL	PRT-IL	TERMCTR	TERM+14d
**Gestation**	Preterm	Preterm	Preterm	Term	Term
**N**	6	5	8	6	3
**Male/Female**	3/3	1/4	4/4	2/4	1/2
**Birth Weight (g)**	413±33	386±56	393±47	863±137	844±28
**Serum glucose (mg/dl)**	41±2*	81±9	71±3	58±5	64±4

Baseline demographic characteristics of preterm baboon infants euthanized at birth (PRTCTR, n = 6), preterm baboon infants receiving 14 days of parenteral nutrition with Intralipids (PRT+IL, n = 5), preterm baboon infants receiving 14 days of parenteral nutrition without Intralipids (PRT-IL, n = 8), term baboon infants euthanized shortly after birth (TERMCTR, n = 6) and term baboon infants survived for 14 days (TERM+14d, n = 3) are shown. Birth weight and serum glucose are expressed as mean ± SEM.

*, p<0.05 PRTCTR vs PRT+IL and PRT-IL (One-way ANOVA with Bonferroni Post-Hoc).

### Liver function tests

**Fig 1:**
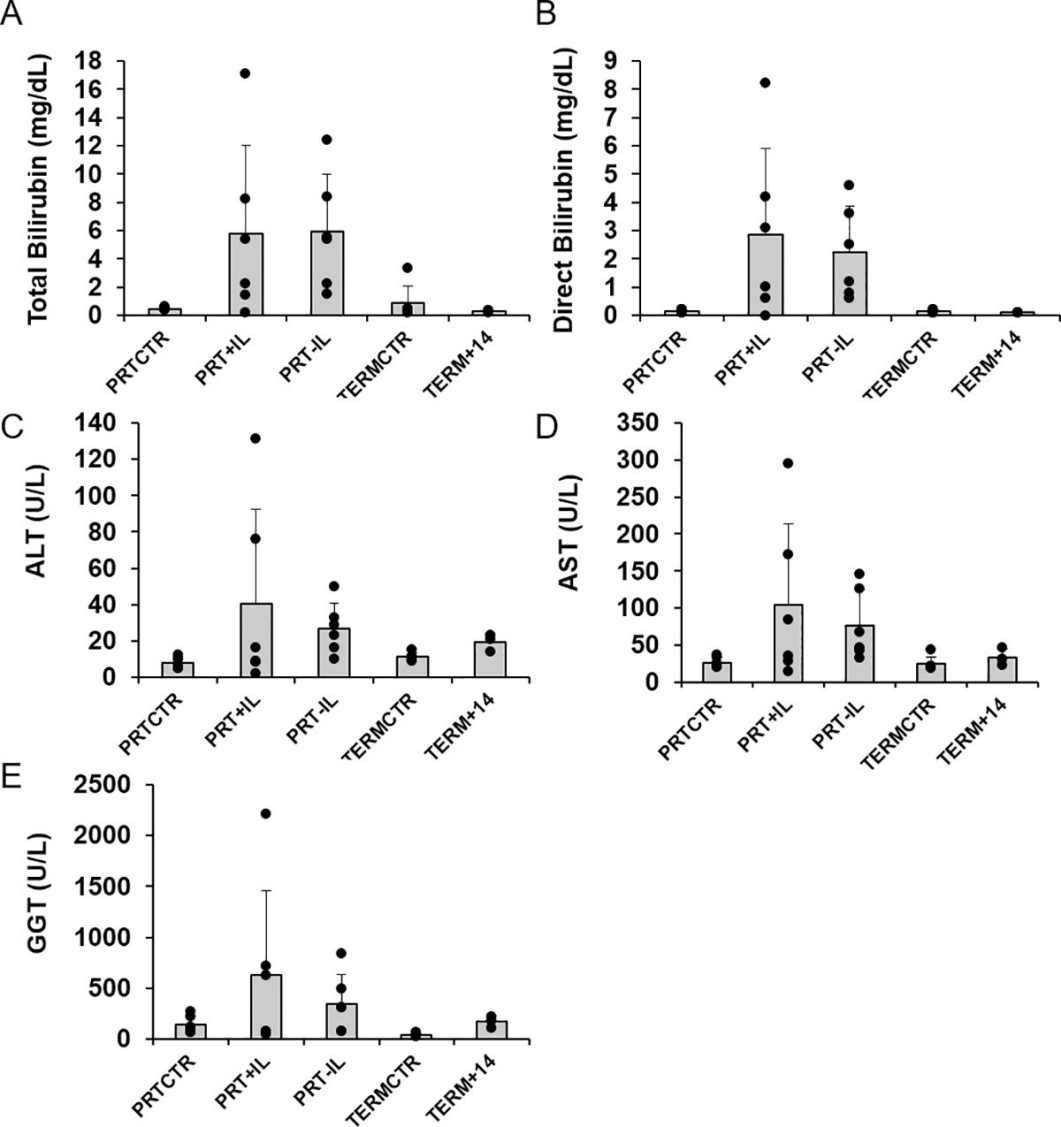
Serum levels of traditional liver function tests in infant baboons. Mean serum [A] Total Bilirubin (in mg/dL), [B] Direct Bilirubin (in mg/dL), [C] Alanine aminotransferase (ALT, in U/L), [D] Aspartate aminotransferase (AST, in U/L) and [E] Gamma-glutamyl transferase (GGT, in U/L) are shown for each group. Error bars represent ± SD. *, P<0.05, significance is based on one-way ANOVA with Bonferroni Post-Hoc. PRTCTR: preterm gestational control, n = 6; PRT+IL: preterm with Intralipids, n = 6; PRT-IL: preterm without Intralipids, n = 6; TERMCTR: term gestational control, n = 6; TERM+14d: term gestational control surviving 14 days, n = 3.

Variability in liver function test results was high for all tests in PRT+IL and PRT-IL. Total Bilirubin and direct bilirubin were significantly different across treatment groups (F = 3.53, p = 0.023 and F = 3.60, p = 0.021, respectively), however we were not able to detect a significant difference between groups. Both total and direct bilirubin tended to be elevated in the PRT+IL and PRT-IL groups compared to PRTCTR and were similar between the TERMCTR and TERM+14d groups (Fig 1A). ALT, AST, and GGT also tended to be higher in the PRT+IL and PRT-IL groups compared to PRTCTR, and were again similar between the TERMCTR and TERM+14d groups, but this was not statistically significant (p = 0.23, p = 0.09, and p = 0.18, respectively; Fig 1C, 1D and 1E).

### ELISA and ELF scores

**Fig 2:**
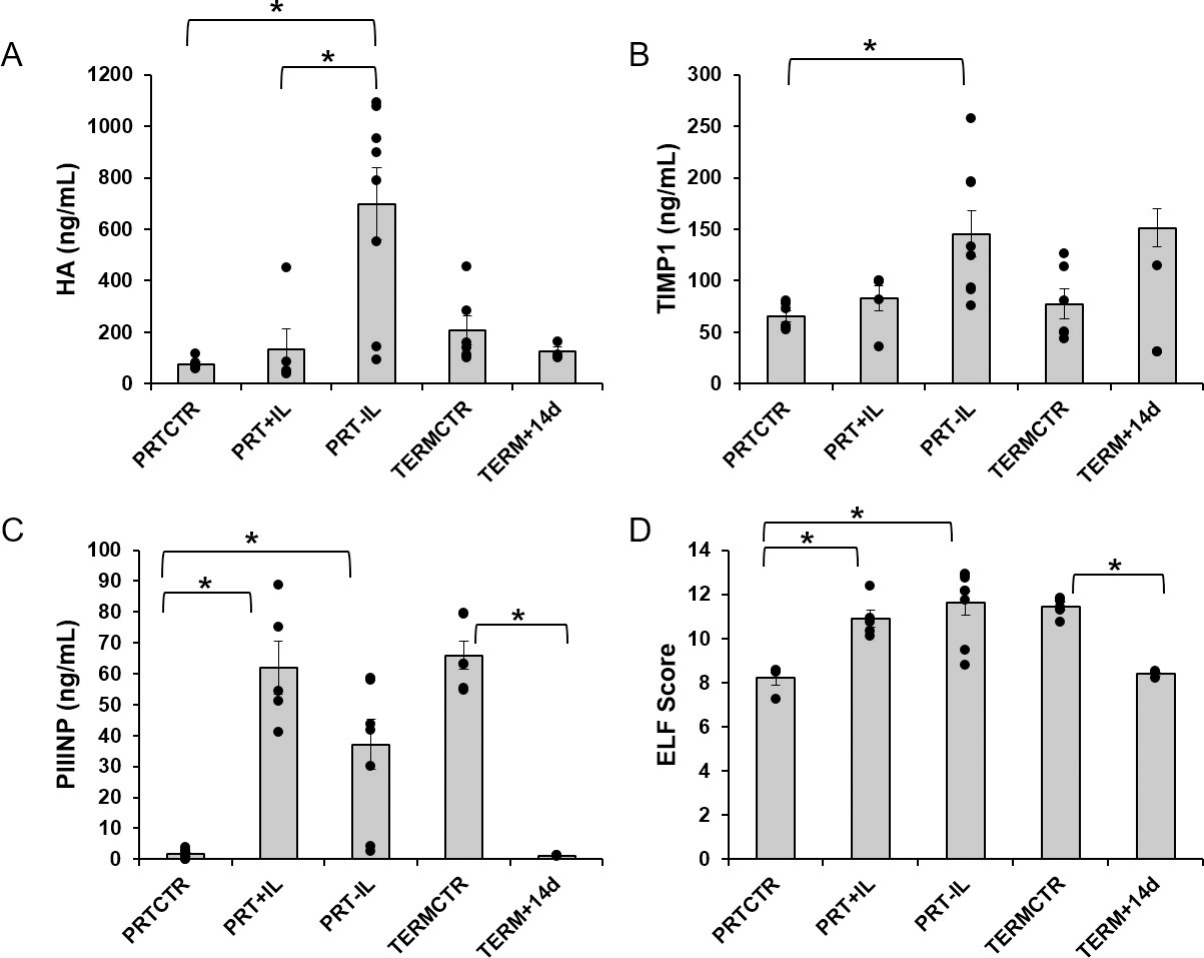
Serum markers of liver fibrosis in infant baboons. Mean [A] Hyaluronic Acid (HA, in ng/mL), [B] TIMP metallopeptidase inhibitor 1 (TIMP1, in ng/mL), and [C] Amino-terminal Propeptide of Type-III Collagen (PIIINP, in ng/mL) levels are shown for each group. [D] Mean Enhanced Liver Fibrosis score (ELF Score) is shown for each group. Error bars represent ± SD. *, P<0.05, significance is based on one-way ANOVA with Bonferroni Post-Hoc. PRTCTR: preterm gestational control, n = 6; PRT+IL: preterm with Intralipids, n = 5; PRT-IL: preterm without Intralipids, n = 8; TERMCTR: term gestational control, n = 6; TERM+14d: term gestational control surviving 14 days, n = 3.

The results of the ELISAs and ELF scores are summarized in Fig 2 and Table 2. Serum HA was significantly higher in the PRT-IL compared to PRT+IL and PRTCTR (p<0.05, Fig 2A). Serum HA was similar between PRTCTR and PRT+IL (p = 1.0) as well as between TERMCTR and TERM+14d (p = 1.0). TIMP1 was 2.2-fold higher in the PRT-IL group compared to the PRTCTR group (p<0.01) but was similar to the PRT+IL group (p = 0.092; Fig 2B). TIMP1 was also similar between the TERMCTR and TERM+14d groups (p = 0.86). PIIINP was significantly higher in the PRT+IL and PRT-IL groups compared to the PRTCTR group (p<0.05) and was similar between the PRT+IL and PRT-IL groups (p = 0.10). PIIINP was significantly lower in the TERM+14d group compared to the TERMCTR group (p<0.05). Similarly, ELF scores were significantly higher in the PRT+IL and PRT-IL groups compared to the PRTCTR group (p<0.05) and were similar between the PRT+IL and PRT-IL groups (p = 1.0, Fig 2D). ELF scores were also significantly lower in the TERM+14d group compared to the TERMCTR group (p<0.05).

**Table 2:** Serum markers of liver fibrosis in infant baboons.

	PRTCTR	PRT+IL	PRT-IL	TERMCTR	TERM+14d
**HA (ng/mL)**	76 ± 9	133 ± 80*	699 ± 141*	208 ± 56	125 ± 20
**TIMP1 (ng/mL)**	66 ± 5	83 ± 12	146 ± 23*	77 ± 15	151 ± 19
**PIIINP (ng/mL)**	1.6 ± 0.7	62 ± 9*	37 ± 8*	66 ± 5	1.1 ± 0.1**
**ELF Score**	8.2 ± 0.3	10.9 ± 0.4*	11.6 ± 0.6*	11.5 ± 0.2	8.4 ± 0.2**

Concentrations of serum markers of liver fibrosis in preterm baboon infants euthanized at birth (PRTCTR, n = 6), preterm baboon infants receiving 14 days of parenteral nutrition with Intralipids (PRT+IL, n = 5), preterm baboon infants receiving 14 days of parenteral nutrition without Intralipids (PRT-IL, n = 8), term baboon infants euthanized shortly after birth (TERMCTR, n = 6), and term baboon infants survived for 14 days (TERM+14d, n = 3) are shown. HA: Hyaluronic Acid, TIMP1: TIMP metallopeptidase inhibitor 1, PIIINP: Amino-terminal Propeptide of Type-III Collagen, ELF: Enhanced Liver Fibrosis score.

*, p<0.05, PRT+IL and/or PRT-IL vs PRTCTR

**, p<0.05, TERM+14d vs TERMCTR. P-values are based on one-way ANOVA with Bonferroni post-hoc. Results are expressed as mean±SEM.

In the present study, the area under the receiver operating characteristic curve was low (<0.5) for all serum biomarkers of liver disease in discriminating fibrosis level. TIMP1 had the greatest area (0.43), followed by ALT (0.37), GGT (0.36), HA (0.35), and ELF score (0.30); direct bilirubin (0.29) and AST (0.18) had the lowest AUCs. TIMP1 also had the greatest ability to discriminate microvesicular steatosis among the serum biomarkers (AUC: 0.48). The Spearman’s correlation coefficients between TIMP1 and markers of liver disease were low and nonsignificant (fibrosis: r_s_ = 0.14, p = 0.40; microvesicular steatosis: r_s_ = 0.14, p = 0.43; and Kupffer cell hypertrophy: r_s_ = 0.18, p = 0.29). The Spearman’s correlation coefficients between HA and markers of liver disease were somewhat higher, but remained nonsignificant (fibrosis: r_s_ = 0.25, p = 0.14; microvesicular steatosis: r_s_ = 0.28, p = 0.1; and Kupffer cell hypertrophy: r_s_ = 0.45, p = 0.07). Finally, Spearman’s correlation coefficient between ELF score and makers of liver disease were highest and some reached significance (fibrosis: r_s_ = 0.34, p = 0.05; microvesicular steatosis: r_s_ = 0.56, p = 0.001; and Kupffer cell hypertrophy: r_s_ = 0.35, p = 0.05).

### Histology and morphometric analyses

**Fig 3:**
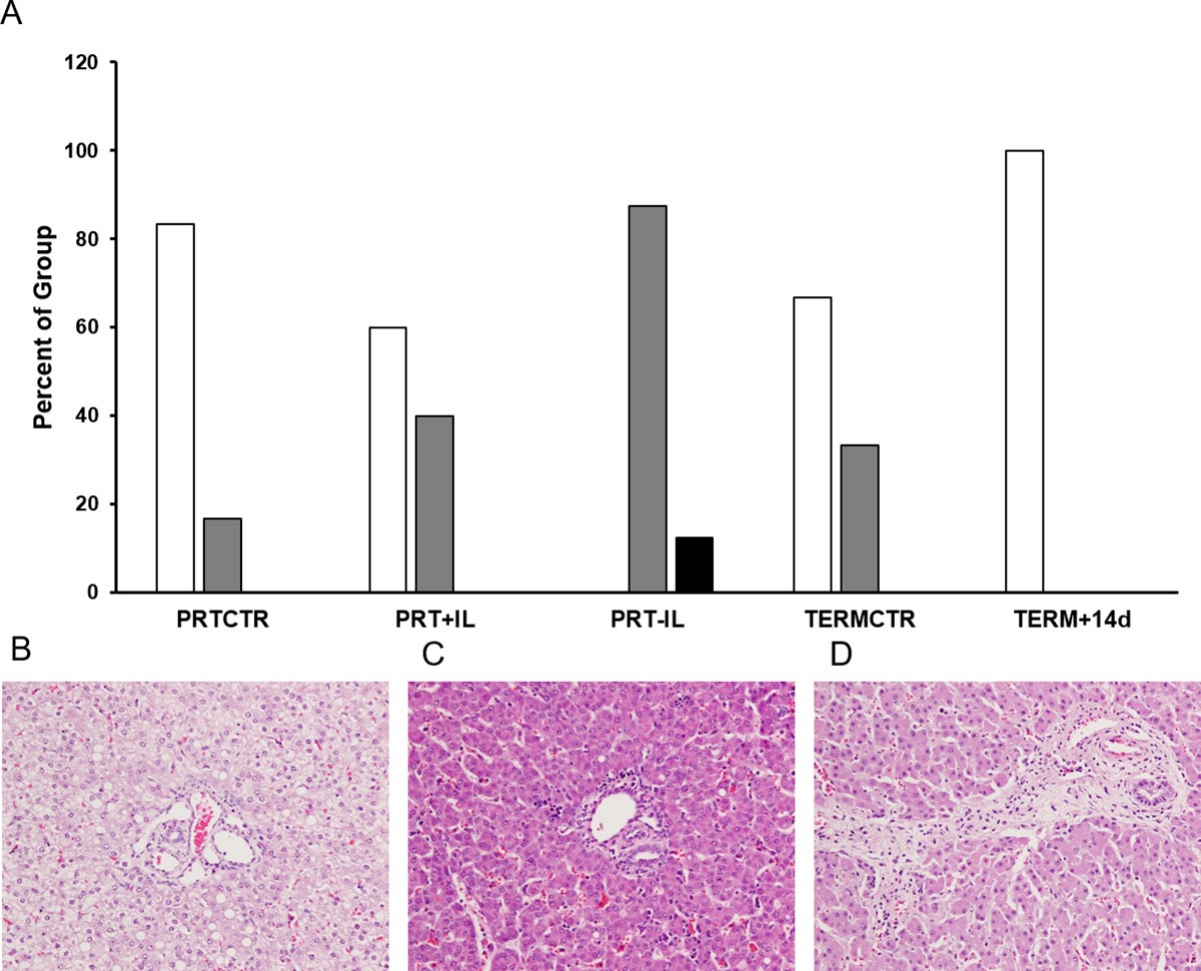
Hepatic fibrosis in infant baboons. [A] Percentage of baboons per group with stage 0 (white bars), stage 1 (grey bars), and stage 2 (black bars) hepatic fibrosis are shown. Trend towards increased fibrosis in PRT-IL group vs PRTCTR (p = 0.003; Kruskal-Wallis test). [B-D] Representative H&E images of each fibrosis score are also shown. Magnification is 20X for each image. [B] Fibrosis score of 0 is shown. [C] Fibrosis score of 1 is shown. [D] Fibrosis score of 2 is shown. PRTCTR: preterm gestational control, n = 6; PRT+IL: preterm with Intralipids, n = 5; PRT-IL: preterm without Intralipids, n = 8; TERMCTR: term gestational control, n = 6; TERM+14d: term gestational control surviving 14 days, n = 3.

The results of the liver histopathology studies are summarized in Fig 3 and Table 3. Outputs of the statistical analysis on the histopathology scores are summarized in Table 4. Representative images for each CD68 score and CK7 score are shown in S1 Fig. Although fibrosis was significantly different across groups (X^2^(4) = 14.49, p = 0.006, Table 4) we were not able to detect a statistically significant difference in level of fibrosis between groups. Fibrosis tended to be increased in the PRT-IL baboons, however, this did not reach adjusted statistical significance (p = 0.003; Fig 3, Tables 3 and 4). Similarly, bile stasis, Kupffer cell hemophagocytosis, and Kupffer cell iron storage were significantly different across groups, but no significant differences between groups were detected (Table 4). Hepatic erythroid hematopoiesis was increased in the PRTCTR group as compared to the PRT+IL group (p = 0.001, Tables 3 and 4). Portal myelopoiesis was also increased in the PRTCTR as compared to the PRT+IL group (p = 0.001, Tables 3 and 4). Lobular myelopoiesis tended to be increased in the PRTCTR compared to the PRT+IL group, but did not reach adjusted significance (p = 0.02, Tables 3 and 4) Kupffer cell hypertrophy was significantly elevated in PRT-IL compared to PRTCTR (p<0.001, Tables 3 and 4). Microvesicular hepatocyte steatosis was significantly increased in the PRT-IL group compared to PRTCTR (p = 0.002, Tables 3 and 4). Hepatocyte iron storage, ongoing portal track development, acinar bile ducts, macrovesicular hepatocyte steatosis, hepatocyte apoptotic bodies, lobular atrophy, CK7 scores, and CD68 scores were not significantly different across groups (Tables 3 and 4). All liver histopathology scores were also similar between the TERMCTR and TERM+14d groups (Tables 3 and 4).

**Table 3:** Liver histopathology scores in infant baboons.

	PRTCTR	PRT+IL	PRT-IL	TERMCTR	TERM+14d
**Fibrosis**					
Portal fibrosis stage [0–4]	0 (0)	0 (0.25)	1 (0)	0 (0.75)	0 (0)
**Extramedullary Hematopoiesis**					
Hepatic erythroid hematopoiesis [0–2]	2 (0)	1 (1)	1.5 (1.25)	0.5 (1)	0 (0.5)
Portal myelopoiesis [0–3]	3 (0)	1 (1.25)	2 (1)	1 (0.75)	1 (0.5)
Lobular myelopoiesis [0–3]	3 (0)	2 (0.75)	2.5 (1)	1 (0)	1 (0.5)
**Kupffer Cells**					
Hypertrophy [0–3]	0 (0)	1 (1.25)	1.5 (1)	1 (0)	1 (0.5)
Hemophagocytosis [0–3]	0 (0)	1 (1.25)	1.5 (1.5)	0 (0)	0 (0.5)
Iron storage [0–3]	0 (0.75)	1.5 (1)	1 (0.5)	0 (0.25)	0 (0.5)
CD68 Score [0–3]	1 (0)	1 (0)	1.5 (1.5)	1 (1)	1 (0)
**Hepatocytes**					
Macrovesicular Steatosis [0–3]	0 (0)	1 (0.5)	0 (0)	0 (0)	0 (0.5)
Microvesicular Steatosis [0–3]	0 (0)	1 (0.25)	1 (1)	1.5 (1)	0 (0.5)
Apoptotic Bodies [0–3]	0 (0)	0 (0.25)	0 (0)	0 (0)	0 (0)
Lobular Atrophy [0–3]	0 (0)	0 (0)	0 (0)	0 (0)	0 (0)
Iron storage [0–3]	1 (0)	1 (0)	1.5 (1.25)	1 (0)	0 (0.5)
**Biliary Tract**					
Bile Stasis [0–3]	0 (0)	1 (1.25)	0 (0.25)	0 (0)	0 (0)
Ductular Proliferation [0–3]	0 (0)	0 (0)	0 (0.25)	0 (0)	0 (0)
CK7 Score [0–3]	0 (0)	1 (1)	1 (1)	1 (1)	1 (1)
Acinar bile ducts (% portal tracts)	43 (30)	20 (29)	43 (19)	23 (16)	50 (20)

Liver histopathology scores in preterm baboon infants euthanized at birth (PRTCTR, n = 6), preterm baboon infants receiving 14 days of parenteral nutrition with Intralipids (PRT+IL, n = 5), preterm baboon infants receiving 14 days of parenteral nutrition without Intralipids (PRT-IL, n = 8), term baboon infants euthanized shortly after birth (TERMCTR, n = 6), and term baboons survived for 14 days (TERM+14d, n = 3) are shown. Histopathology measures are followed by their respective scoring ranges in square brackets, unless otherwise indicated. Results are reported as Median (IQR).

**Table 4:** Statistical analysis of liver histopathology scores.

	FIBP	EMHE	EMHP	EMHL	KCH	KCHP	IRONK	CD68	HEPSM	HEPSMI	HEPA	HEPLA	IRONH	BILES	DUCTP	CK7	ACBILD
**Chi-Square**	14.49	14.22	17.16	16.4	13.61	10.07	13.69	4.71	9.28	14.14	2.65	2.5	2.84	13.17	5.19	4.1	N/A
**Df**	4	4	4	4	4	4	4	4	4	4	4	4	4	4	4	4	N/A
**p-value**	0.006	0.007	0.002	0.003	0.009	0.039	0.008	0.319	0.06	0.007	0.618	0.645	0.585	0.01	0.268	0.392	0.064
**PRTCTR vs PRT+IL**	0.264	0.103	0.001*	0.022	0.027	0.052	0.012	N/A	N/A	0.007	N/A	N/A	N/A	0.014	N/A	N/A	N/A
**PRTCTR vs PRT-IL**	0.003	0.051	0.066	0.1	0.0004*	0.007	0.028	N/A	N/A	0.002*	N/A	N/A	N/A	0.207	N/A	N/A	N/A
**PRT+IL vs PRT-IL**	0.029	0.42	0.036	0.179	0.125	0.268	0.282	N/A	N/A	0.478	N/A	N/A	N/A	0.058	N/A	N/A	N/A
**TERMCTR vs TERM+14d**	0.22	0.041	0.443	0.365	0.293	0.432	0.365	N/A	N/A	0.047	N/A	N/A	N/A	0.5	N/A	N/A	N/A

Statistical analysis of liver histopathology scores in preterm baboon infants euthanized at birth (PRTCTR, n = 6), preterm baboon infants receiving 14 days of parenteral nutrition with Intralipids (PRT+IL, n = 5), preterm baboon infants receiving 14 days of parenteral nutrition without Intralipids (PRT-IL, n = 8), term baboon infants euthanized shortly after birth (TERMCTR, n = 6), and term baboons survived for 14 days (TERM+14d, n = 3) are shown. Significance based on Kruskall-Wallis Test with Post-Hoc, except for ACBILD, which is based on One-way ANOVA with Bonferroni Post-Hoc. p<0.05 was considered significant for Kruskall-Wallis, with Post-Hoc significance adjusted to p<0.0025 and p<0.05 was considered significant for ANOVA. FIBP: portal fibrosis stage, EMHE: hepatic erythroid hematopoiesis, EMHP: portal myelopoiesis, EHML: lobular myelopoiesis, KCH: Kupffer cell hypertrophy, KCHP: Kupffer cell hemophagocytosis, IRONK: Kupffer cell iron storage, CD68: CD68 score, HEPSM: Hepatocyte macrovesicular steatosis, HEPSMI: Hepatocyte microvesicular steatosis, HEPA: Hepatocyte apoptotic bodies, HEPLA: Hepatocyte lobular atrophy, IRONH: Hepatocyte iron storage, BILES: bile stasis, DUCTP: ductular proliferation, CK7: CK7 score, ACBILD: acinar bile ducts.

### RT-PCR

**Fig 4:**
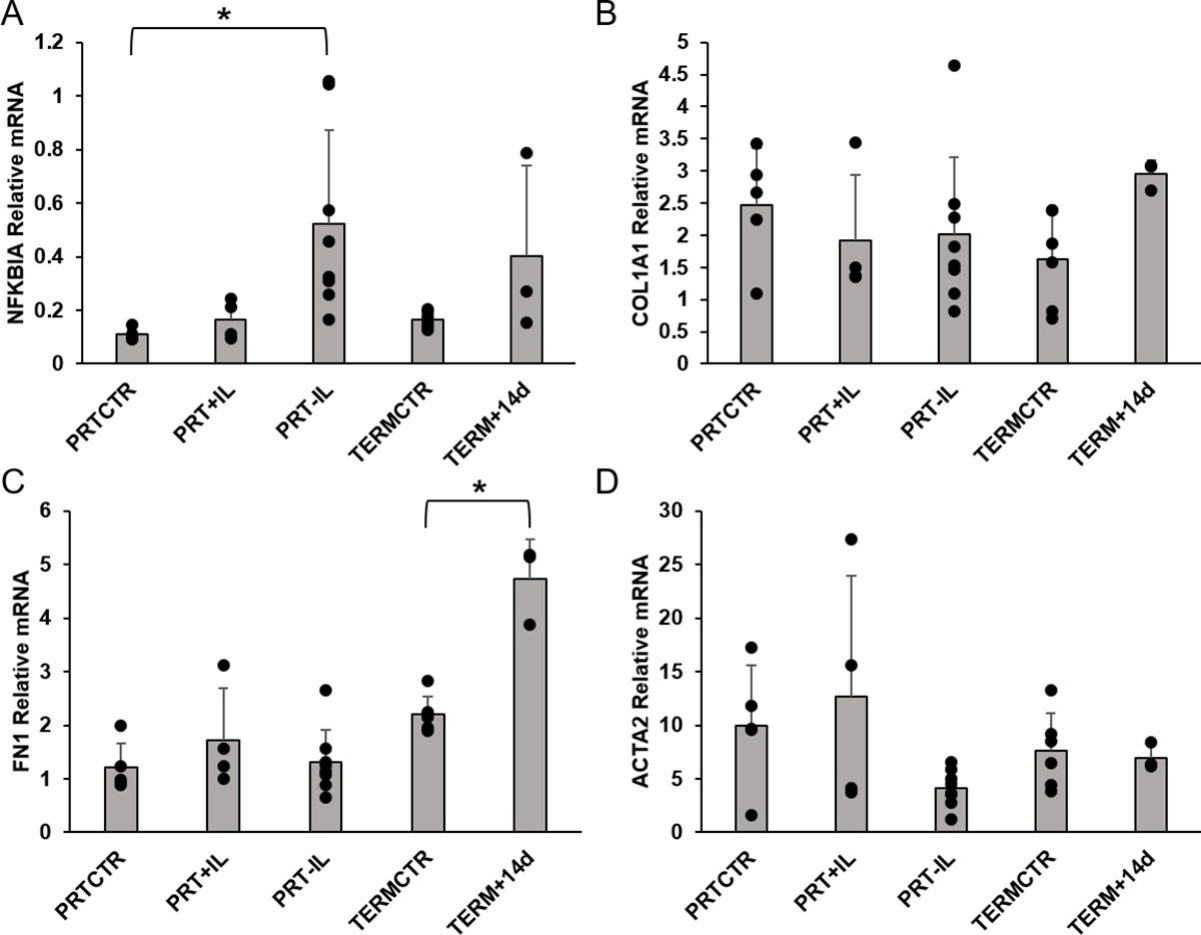
Liver mRNA expression of Pro-fibrotic and Pro-inflammatory genes. Relative mRNA expression of [A] NFKBIA, [B] COL1A1, [C] FN1, and [D] ACTA2 in the livers of infant baboons are shown. *, p<0.05. PRTCTR: preterm gestational control, n = 5; PRT+IL: preterm with Intralipids, n = 4; PRT-IL: preterm without Intralipids, n = 8; TERMCTR: term gestational control, n = 6; TERM+14d: term gestational control surviving 14 days, n = 3.

The results of the RT-PCR analysis are summarized in Fig 4. Relative mRNA expression of NFKBIA was significantly increased in PRT-IL animals compared to PRTCTR (p = 0.045), but was similar between PRT-IL and PRTCTR (p = 1.0) and between PRT-IL and PRT+IL (p = 0.18) animals (Fig 4A). Relative mRNA expression of NFKBIA was also similar between TERMCTR and TERM+14d animals (p = 1.0). Relative mRNA expression of COL1A1 was similar between groups (p = 0.34, Fig 4B). Relative mRNA expression of FN1 tended to be higher in term animals compared to preterm animals but was not significantly different among the preterm animals (p = 1.0). Expression of FN1 was significantly higher in TERM+14d animals compared to TERMCTR animals (p<0.01, Fig 4C). Relative mRNA expression of ACTA2 was not significantly different between groups (p = 0.12, Fig 4D). Finally, correlations between NFKBIA and PIIINP were found (r_s_ r 0.44, p = 0.02) whereas no correlations were found with TIMP1, HA and ELF score (p = 0.1 for all).

## Discussion

Parenteral-nutrition associated liver disease continues to be a major source of morbidity and mortality among critically-ill neonates; however, techniques to identify and monitor disease progression remain limited. The purpose of this study was to investigate whether several novel markers of liver disease currently utilized in adults and children (HA, TIMP1, PIIINP, ELF) are associated with liver disease in premature baboons receiving PN. This approach has several unique advantages, including the ability to correlate serum levels of HA, TIMP1, and PIIINP with histological evaluation of the liver, without the need for risky biopsies or the use of autopsy samples, which may not accurately represent the pathology of liver disease in surviving infants. Furthermore, this approach allows for the inclusion of fetal and term control animals that are well-matched to the study animals to provide further context for data interpretation. Finally, this approach allowed for the inclusion of animals exposed to PN with and without IL, providing an opportunity to better understand the role of lipid infusions in the development of liver disease.

Histopathologic and RT-PCR analysis of the livers of premature baboons treated with PN for 14 days revealed limited evidence of fibrosis or inflammation in either IL group. However, given the two week time constraint of our model, we wouldn’t necessarily expect to find more pronounced evidence of liver disease. Nevertheless, both preterm baboon groups exhibited some degree of liver disease. Fibrosis was highest in the PRT-IL baboons, although this did not reach statistical significance (Fig 3). Fibrosis was lower in PRT+IL baboons compared to PRT-IL baboons, but still slightly higher than the controls. Measures of hepatocyte and Kupffer cell damage also tended to be higher in the preterm baboon groups compared to the control groups, although there was high variability and most of these differences were not significant (Tables 3 and 4). Ductular reaction and CD68+ macrophage staining tended to be more pronounced in PRT-IL and PRT+IL compared to PRTCTR, but these trends also did not reach significance. mRNA expression of NFKBIA was significantly higher in PRT-IL animals compared to controls, but other mRNA markers of inflammation and fibrosis were similar between preterm groups. We speculate that the relatively mild liver disease seen is due predominantly to the short duration of the PN exposure but perhaps the significant increase in NFKBIA expression in PRT-IL animals, which is well known for its involvement in inflammatory responses and induction of apoptosis, may represent the potential early mechanism leading to fibrosis after prolonged exposures [30]. Indeed, in a previous study conducted by our group, in which baboons were kept on PN with IL for 3–4 weeks, the animals exhibited more advanced fibrosis (up to stage 3) [22]. We were surprised to find that the TERMCTR group exhibited relatively high levels of fibrosis- only slightly lower than the PRT+IL group. Whether this finding represents normal postnatal development or whether the insulin clamps the animals received contributed to these findings remains unclear. On the other hand, these histopathological findings may represent some inaccuracy of methods to evaluate early stages of fibrosis since in this study, an experienced blinded pathologist analyzed the data. We recognize that these factors represent serious limitations to the ability to associate serum markers of liver disease with histopathologic levels of liver disease; nevertheless, this study offers valuable insight into the pathology of neonatal liver disease.

HA is a glycosaminoglycan present in synovial fluid in joints and in some tissues, such as the liver. It is often elevated with chronic liver disease due to increased production by hepatic stellate cells and decreased clearance by sinusoidal endothelial cells. It has been found to correlate with stage of liver fibrosis in children with NAFLD, cystic fibrosis, and biliary atresia and in adults with chronic hepatitis C and alcoholic liver disease [16,31–36]. Consistent with the relatively higher levels of fibrosis seen in the PRT-IL group, serum levels of HA were significantly higher in this group compared to all other groups. Although both the PRT+IL and TERMCTR groups also exhibited some fibrosis, HA levels were similar between these groups and the PRTCTR and TERM+14d groups, which exhibited limited to no fibrosis. HA levels did not exhibit correlation with fibrosis, steatosis, or Kupffer cell hypertrophy; we are not surprised, as adverse histopathological changes may need more time. These findings suggest HA may not be predictive of early fibrosis but is better than or equal to traditional markers of liver disease.

Hepatic stellate cells express TIMP1 in later phases of liver injury to modify expression of matrix metalloproteinases and decrease matrix degradation [37,38]. TIMP1 has been shown to be elevated in adult populations with hepatic fibrosis due to chronic hepatitis C, Alcoholic Liver Disease, and NAFLD, and in children with liver disease secondary to cystic fibrosis [38–42]. Moreover, it rapidly decreases once the underlying insult to the liver is stopped. Similar to HA, serum levels of TIMP1 were generally higher in the PRT-IL group, though this difference was only significant when compared to the PRTCTR group. TIMP1 levels tended to be similar across the remaining groups, with the exception of the TERM+14d group, which exhibited somewhat elevated TIMP1 levels, though with high variability. Although TIMP1 had the greatest ability of the markers examined here to discriminate fibrosis when compared to other novel and traditional markers of liver disease, TIMP1 still did not exhibit as strong a correlation with any of the histopathological findings. These findings suggest TIMP1 may not be a sensitive marker of early neonatal liver disease but perhaps further studies with more advanced disease will lead to higher sensitivity.

PIIINP is created during the conversion of procollagen III to collagen III, an important contributor to liver fibrosis [43]. Studies have suggested serum levels of PIIINP correlate with fibrosis in alcoholic liver disease, hepatitis C and NAFLD [15]. We found that PIIINP was highest shortly after birth at term but was significantly lower when born preterm (PRTCTR vs TERMCTR). After 14 days, PIIINP increased significantly in both the PRT+IL and PRT-IL groups, whereas it decreased significantly in the TERM+14d group. Interestingly, PIIINP was lower in the PRT-IL group (the group with the greatest fibrosis) compared to both PRT+IL and TERMCTR (both groups with relatively less fibrosis). These results are in agreement with early studies that suggested PIIINP levels may decrease with increasing disease [43], however, other studies have not replicated these findings. PIIINP mildly correlated with increases in the NFKBIA expression in the liver, which may be representative of the early stages of inflammation. Our results suggest PIIINP may be a reasonable indicator of liver disease after the immediate post-natal period, but shortly after birth at term, PIIINP may be naturally elevated. Furthermore, PIIINP may be most informative during the earlier stages of liver damage, and less informative as fibrosis progresses.

The ELF score uses an algorithm combining HA, TIMP1, and PIIINP to create a score that has been found to be highly sensitive and specific for determining stage of liver fibrosis in adults and pediatric patients with NAFLD, and is under investigation for cystic fibrosis liver disease and biliary atresia [19,25,44–47]. The ELF score was significantly lower in the PRTCTR and TERM+14d groups vs all other groups, but was similar between the PRT+IL, PRT-IL, and TERMCTR groups. All ELF scores outside of the PRTCTR and TERM+14d groups were above the value for suspected liver fibrosis established in pediatric populations ([25]; proposed cut offs: any fibrosis: 8.49, mild fibrosis: 9.33, moderate fibrosis: 9.54, significant fibrosis: 10.18, advanced fibrosis: 10.51). Finally, ELF scores exhibited the greatest correlation between fibrosis levels, steatosis and Kupffer cell hypertrophy of the markers examined in this study. However, the significance of these findings are difficult to interpret, as ELF scores in term controls were similar to those of the PRT+IL and PRT-IL animals. Whether the increases in ELF scores seen here are due simply to normal postnatal maturation cannot be determined, as there are currently no standard scoring values established in neonates. This highlights the importance of establishing “normalcy” values in future studies.

The pathogenesis of PNALD is multifactorial and remains poorly understood [48]. It has often been proposed that the soy-based IL used in standard PN is a primary source of injury due to the development of biliary stasis [49]. However, other work has argued that fatty acid deficiency plays a major role in the development of liver disease [50,51]. Our findings of greater liver damage, as well as higher HA and TIMP1, in the PRT-IL group of baboons suggest that nutrient deficiency may be an important contributor to neonatal liver disease. The mechanism for lack of nutrients causing liver disease has been previously described in non-alcoholic liver disease via autophagy [52]. This is a highly conserved intracellular process for the degradation and recycling of cellular components to provide amino acids, glucose and free fatty acids where endothelial cells, macrophages and hepatic stellate cells also employ autophagy [52]. These findings are especially clinically relevant, as many NICUs significantly limit the amount of IL given to infants with early developing signs of TPN cholestasis [53,54]. However, we also noted somewhat greater prevalence of biliary stasis in the PRT+IL group, and although this was not significantly elevated compared to the other groups, this may be due to the relatively short exposure time, rather than any protective effect of intralipids for liver disease. Of note, among the traditional markers of liver disease examined (Total bilirubin, direct bilirubin, ALT, AST, GGT) only total and direct bilirubin were significantly elevated with evidence of liver fibrosis. Levels of ALT, AST, and GGT were almost entirely within normal limits and demonstrated no significant differences between groups. The normal liver function test levels observed in animals with histological fibrosis further highlights the limited utility of these traditional markers for determining hepatic fibrosis and emphasizes the need for more accurate markers.

In addition to intravenous lipid formulation, other nutritional factors have also been implicated in the development of liver disease in the neonatal period, including excessive amounts of trace elements, such as copper and manganese. In this study, all preterm animals received the exact same formulation and amount of trace elements according to a standardized protocol. Furthermore, these animals were only exposed to PN for 14 days; therefore, it seems unlikely that the trace elements would have built to toxic levels in such a short amount of time. However, we did not test different doses of trace elements, and the exact dose and level of each trace element required in the neonatal period and their possible roles in liver disease remain to be determined. Parenteral amino acids are tolerated in premature and term infants receiving PN up to 3.5 g/kg/day for prolonged periods, which is what our PRT-IL and PRT+IL animals received. Historically, increased administration of parenteral amino acids was believed to be associated with earlier onset and increased severity of liver disease, decreased bile flow, and hepatotoxicity due to accumulation of specific amino acids (methionine, tryptophan) and photo-degradation products of amino acids [55]. However, more recent data have not supported these findings [56]. Furthermore, in human studies, when IL are limited but amino acids are continued, the currently used markers for liver disease return back towards baseline [53]. However, we cannot be certain amino acid solutions do not contribute to liver disease as both animals that received PN had some evidence of injury and we did not test one group without amino acid solution. On the other hand, since the dose of amino acids was the same in both groups of preterm chronically ventilated animals along with evidence of hepatic fibrosis in murine models when no supplemental IL or enteral feeds are provided supports our findings [50].

This study has several important limitations that need to be taken into consideration. First, as previously described, the duration of PN therapy received by the animals in this study was relatively short and, as a result, the liver disease seen was fairly mild. There are two main reasons for this study design. First, due to the severity of illness, survival in these extremely premature animals beyond 2 weeks is very limited. Second, the animals included in this study served as shared controls for ongoing studies, all of which required the 2-week endpoint (and also required the insulin clamp in the TERMCTR group). An additional limitation to this study is the small sample, which contributed to the larger than expected variability in levels of serum markers and histopathology scores within the test groups. As previously discussed, all animals included in these experiments were shared controls; therefore, we are unable to include more animals. Furthermore, given the study design restrictions previously discussed (the fact that we were unlikely to be able to achieve a greater degree of liver disease in our study animals) we felt we could not justify running additional animals solely for these experiments.

## Conclusion

Chronically ventilated premature baboons develop signs of liver disease after exposure to 14 days of PN, which are most pronounced in animals exposed to PN without IL. Serum levels of HA and TIMP1 are significantly higher in the same group of animals, and therefore, HA and TIMP1 could potentially be utilized for monitoring early hepatic injury due to PNALD in neonates. Future studies including larger numbers of neonates are warranted, given the importance of protecting the developing liver from injury.

## Supporting information

S1 Fig Representative images of CD68 and CK7 scoring. Representative images for each CD68 score and CK7 score are shown. Magnification is 20X for all images. [A] CD68 score of 0 is shown. No CD68+ macrophages are seen. [B] CD68 score of 1 is shown. Few scattered CD68+ macrophages are seen. [C] CD68 score of 2 is shown. A moderate number of CD68+ macrophages are seen. [D] CD68 score of 3 is shown. Marked infiltration of CD68+ macrophages is seen. [E] CK7 score of 0 is shown. No CK7+ progenitor cells are seen around this portal tract. [F] CK7 score of 1 is shown. Few single CK7+ progenitor cells are seen around this portal tract. [G] CK7 score of 2 is shown. Several clusters of CK7+ progenitor cells are seen around this portal tract.
(TIF)

## Abbreviations

ACTA2: Actin Alpha 2, smooth muscle
ALT: alanine aminotransferase
AST: aspartate aminotransferase
C/S: Cesarean section
COL1A1: Collagen Type 1 alpha 1
ELF: Enhanced Liver Fibrosis Score
FN1: Fibronectin 1
GGT: Gamma-glutamyl transferase
HA: Hyaluronic Acid
IL: Intralipid
NAFLD: Non-Alcoholic Fatty Liver Disease
NFKBIA: NFKB inhibitor alpha
PIIINP: Amino-terminal Propeptide of Type-III Collagen
PN: Parenteral Nutrition
PNALD: Parenteral Nutrition-Associated Liver Disease
PRT+IL: preterm baboon receiving parenteral nutrition with intralipids
PRTCTR: preterm gestational control baboon
PRT-IL: preterm baboon receiving parenteral nutrition without intralipids
TBRI: Texas Biomedical Research Institute
TERMCTR: term gestational control baboon
TIMP1: TIMP Metallopeptidase Inhibitor 1
UTHSCSA: University of Texas Health Science Center at San Antonio

## References

[1] Kelly DA. Liver complications of pediatric parenteral nutrition—epidemiology. Nutrition. 1998;14(1):153–7. 10.1016/s0899-9007(97)00232-3 9437702

[2] Feldstein AE, Nobili V. Biomarkers in nonalcoholic fatty liver disease: a new era in diagnosis and staging of disease in children. J Pediatr Gastroenterol Nutr. 2010;51(4):378–9. 10.1097/MPG.0b013e3181ecf3d4 20808243 PMC2950320

[3] Shah AG, Lydecker A, Murray K, Tetri BN, Contos MJ, Sanyal AJ. Comparison of noninvasive markers of fibrosis in patients with nonalcoholic fatty liver disease. Clin Gastroenterol Hepatol. 2009;7(10):1104–12. 10.1016/j.cgh.2009.05.033 19523535 PMC3079239

[4] Castera L. Invasive and non-invasive methods for the assessment of fibrosis and disease progression in chronic liver disease. Best Pract Res Clin Gastroenterol. 2011;25(2):291–303. 10.1016/j.bpg.2011.02.003 21497746

[5] Alisi A, Vito R De, Monti L, Nobili V. Liver fibrosis in paediatric liver diseases. Best Pract Res Clin Gastroenterol. 2011;25:259–68. 10.1016/j.bpg.2011.02.008 21497743

[6] Papastergiou V, Tsochatzis E, Burroughs AK. Non-invasive assessment of liver fibrosis. Ann Gastroenterol. 2012;25:218–31. 24714123 PMC3959378

[7] Poynard T, Ngo Y, Perazzo H, Munteanu M, Lebray P, Moussalli J, et al Prognostic value of liver fibrosis biomarkers: a meta-analysis. Gastroenterol Hepatol. 2011;7(7):445–54. PMC3264893 22298979

[8] Bravo AA, Sheth SG, Chopra S. Liver biopsy. N Engl J Med. 2001;344(7):495–500. 10.1056/NEJM200102153440706 11172192

[9] Ovchinsky N, Moreira RK, Lefkowitch JH, Lavine JE. The liver biopsy in modern clinical practice: a pediatric point-of-view. Adv Anat Pathol. 2012;19(4):250–62. 10.1097/PAP.0b013e31825c6a20 22692288 PMC3404724

[10] Yang HR, Kim HR, Kim MJ, Ko JS, Seo JK. Noninvasive parameters and hepatic fibrosis scores in children with nonalcoholic fatty liver disease. World J Gastroenterol. 2012;18(13):1525–30. 10.3748/wjg.v18.i13.1525 22509085 PMC3319949

[11] Lebensztejn DM, Skiba E, Tobolczyk J, Sobaniec-lotowska ME, Kaczmarski M. Diagnostic accuracy of serum biochemical fibrosis markers in children with chronic hepatitis B evaluated by receiver operating characteristics analysis. World J Gastroenterol. 2005;11(45):7192–6. 10.3748/wjg.v11.i45.7192 16437671 PMC4725072

[12] Dhawan A, Trivedi P, Cheeseman P, Baker AJ, Howard ER, Mieli-vergani G. Serum hyaluronic acid as an early prognostic marker in biliary atresia. J Pediatr Surg. 2001;36(3):443–6. 10.1053/jpsu.2001.21622 11226992

[13] Pereira TN, Lewindon PJ, Smith JL, Murphy TL, Lincoln DJ, Shepherd RW, et al Serum markers of hepatic fibrogenesis in cystic fibrosis liver disease. J Hepatol. 2004;41:576–83. 10.1016/j.jhep.2004.06.032 15464237

[14] Parkes J, Guha IN, Harris S, Rosenberg WMC, Roderick PJ. Systematic review of the diagnostic performance of serum markers of liver fibrosis in alcoholic liver disease. Comp Hepatol [Internet]. 2012;11(1):1 Available from: Comparative Hepatology 10.1186/1476-5926-11-1 23273224 PMC3583674

[15] Rossi E, Adams LA, Bulsara M, Jeffrey GP. Assessing liver fibrosis with serum marker models. Clin Biochem Rev. 2007;28:3–10. 17603636 PMC1904421

[16] Suzuki A, Angulo P, Lymp J, Li D, Satomura S, Lindor K. Hyaluronic acid, an accurate serum marker for severe hepatic fibrosis in patients with non-alcoholic fatty liver disease. Liver Int. 2005;25:779–86. 10.1111/j.1478-3231.2005.01064.x 15998429

[17] Castera L. Non-invasive assessment of liver fibrosis in chronic hepatitis C. Hepatol Int. 2011;5:625–34. 10.1007/s12072-010-9240-0 21484142 PMC3090550

[18] Baranova A, Lal P, Birerdinc A, Younossi ZM. Non-Invasive markers for hepatic fibrosis. BMC Gastroenterol. 2011;11(1):91. 21849046 10.1186/1471-230X-11-91 PMC3176189

[19] Lichtinghagen R, Pietsch D, Bantel H, Manns MP, Brand K, Bahr MJ. The Enhanced Liver Fibrosis (ELF) score: normal values, influence factors and proposed cut-off values. J Hepatol. 2013;59(2):236–42. 10.1016/j.jhep.2013.03.016 23523583

[20] Soden JS, Lovell MA, Brown K, Partrick DA, Sokol RJ. Failure of resolution of portal fibrosis during omega-3 fatty acid lipid emulsion therapy in two patients with irreversible intestinal failure. J Pediatr. 2010;156(2):327–31. 10.1016/j.jpeds.2009.08.033 20105644

[21] Blanco CL, McGill-Vargas LL, McCurnin D, Quinn AR. Hyperglycemia increases the risk of death in extremely preterm baboons. Pediatr Res. 2013;73(3):337–43. 10.1038/pr.2012.184 23364173 PMC4112412

[22] Kerecman J, Mehrotra A, Goodman Z. Liver disease after intensive care of premature baboons: histopathologic observations. J Pediatr Gastroenterol Nutr. 2013;57(2):172–9. 10.1097/MPG.0b013e318293e404 23880624 PMC3738296

[23] Blanco CL, McGill-Vargas LL, Gastaldelli A, Seidner SR, McCurnin DC, Leland MM, et al Peripheral insulin resistance and impaired insulin signaling contribute to abnormal glucose metabolism in preterm baboons. Endocrinology. 2015;156(3):813–23. 10.1210/en.2014-1757 25560831 PMC4330304

[24] Blanco CL, Gastaldelli A, Anzueto DG, Winter LA, Seidner R, Mccurnin DC, et al Effects of intravenous AICAR (5-aminoimidazole-4-carboximide riboside) administration on insulin signaling and resistance in premature baboons, Papio sp. PLoS One. 2018;1–19. 10.1371/journal.pone.0208757 PMC6291136 30540820

[25] Alkhouri N, Carter-Kent C, Lopez R, Rosenberg WM, Pinzani M, Bedgoni G, et al A combination of the pediatric NAFLD fibrosis index and enhanced liver fibrosis test identifies children with fibrosis. Clin Gastroenterol Hepatol. 2011;9(2):150–155.e1. 10.1016/j.cgh.2010.09.015 20888433

[26] French METAVIR Cooperative Study Group. Intraobserver and interobserver variations in liver biopsy interpretation in patients with chronic hepatitis C. Hepatology. 1994;20. 8020885

[27] Bedossa P, Poynard T. An algorithm for the grading of activity in chronic hepatitis C. Hepatology. 1996;24(2):22–6. 10.1002/hep.510240201 8690394

[28] Miranda RN, Omurtag K, Castellani WJ, De las Casas LE, Quintanilla NM, Kaabipour E. Myelopoiesis in the liver of stillborns with evidence of intrauterine infection. Arch Pathol Lab Med. 2006;130(12):1786–91. 10.1043/1543-2165(2006)130[1786:MITLOS]2.0.CO;2 17149951

[29] Eleazar JA, Memeo L, Jhang JS, Mansukhani MM, Chin S, Park SM, et al Progenitor cell expansion: An important source of hepatocyte regeneration in chronic hepatitis. J Hepatol. 2004;41(6):983–91. 10.1016/j.jhep.2004.08.017 15582132

[30] Bai F, Huang Q, Nie J, Lu S, Lu C, Zhu X, et al Trolline ameliorates liver fibrosis by inhibiting the NF-κB pathway, promoting HSC apoptosis and suppressing autophagy. Cell Physiol Biochem. 2017;44:436–446. 10.1159/000485009 29141243

[31] Kobayashi H, Horikoshi K, Yamataka A, Yamataka T, Okazaki T, Lane GJ, et al Hyaluronic acid: a specific prognostic indicator of hepatic damage in biliary atresia. J Pediatr Surg. 1999;34(12):1791–4. 10.1016/s0022-3468(99)90314-7 10626856

[32] Rostami S, Parsian H. Hyaluronic acid: from biochemical characteristics to its clinical translation in assessment of liver fibrosis. Hepat Mon. 2013;13(12):1–9. 10.5812/hepatmon.13787 PMC3877656 24403913

[33] Kaneda H, Hashimoto E, Yatsuji S, Tokushige K, Shiratori K. Hyaluronic acid levels can predict severe fibrosis and platelet counts can predict cirrhosis in patients with nonalcoholic fatty liver disease. J Gastroenterol Hepatol. 2006;21:1459–65. 10.1111/j.1440-1746.2006.04447.x 16911693

[34] Hartley JL, Brown RM, Tybulewicz A, Hayes P, Wilson DC, Gillett P, et al Hyaluronic acid predicts hepatic fibrosis in children with hepatic disease. J Pediatr Gastroenterol Nutr. 2006;43. 10.1097/01.mpg.0000228121.44606.9f 16877988

[35] Nobili V, Alisi A, Torre G, Vito RDE, Pietrobattista A, Morino G, et al Hyaluronic acid predicts hepatic fibrosis in children with nonalcoholic fatty liver disease. Transl Res. 2010;156(4):229–34. 10.1016/j.trsl.2010.05.008 20875899

[36] Hasegawa BT, Sasaki T, Kimura T, Hoki M, Okada A, Mushiake S, et al Measurement of serum hyaluronic acid as a sensitive marker of liver fibrosis in biliary atresia. J Pediatr Surg. 2000;35(11):1643–6. 10.1053/jpsu.2000.18342 11083443

[37] Nie Q, Zhang Y, Xie Y, Luo X, Shao B, Li J, et al Correlation between TIMP-1 expression and liver fibrosis in two rat liver fibrosis models. World J Gastroenterol. 2006;12(19):3044–9. 10.3748/wjg.v12.i19.3044 16718785 PMC4124379

[38] Kossakowska AE, Edwards DR, Lee SS, Urbanski LS, Stabbler AL, Zhang C, et al Altered balance between matrix metalloproteinases and their inhibitors in experimental biliary fibrosis. Am J Pathol. 1998;153(6):1895–902. 10.1016/S0002-9440(10)65703-3 9846979 PMC1866318

[39] Dechene A, Sowa J, Gieseler RK, Jochum C, Bechmann LP, Fouly A El, et al Acute liver failure is associated with elevated liver stiffness and hepatic stellate cell activation. Hepatology. 2010;52(3):1008–16. 10.1002/hep.23754 20684020

[40] Rath T, Menendez K, Kügler M, Hage L, Wenzel C, Schulz R, et al TIMP-1/-2 and transient elastography allow non invasive diagnosis of cystic fibrosis associated liver disease. Dig Liver Dis. 2012;44:780–7. 10.1016/j.dld.2012.04.008 22652148

[41] Fabre V, Wu H, Pondtor S, Coutinho H, Acosta L, Jiz M, et al Tissue inhibitor of matrix-metalloprotease–1 predicts risk of hepatic fibrosis in human Schistosoma japonicum infection. J Infectous Dis. 2011;203:707–14. 10.1093/infdis/jiq099 PMC3072733 21199883

[42] Carlson JJ, Kowdley K V, Sullivan SD, Ramsey SD, Veenstra DL. An evaluation of the potential cost-effectiveness of non-invasive testing strategies in the diagnosis of significant liver fibrosis. J Gastroenterol Hepatol. 2009;24:786–91. 10.1111/j.1440-1746.2009.05778.x 19457153 PMC3676667

[43] Mutimer DJ, Bassendine MF, Kelly P, James OFW. Is measurement of type III procollagen amino propeptide useful in primary biliary cirrhosis? J Hepatol. 1989;9:184–9. 10.1016/0168-8278(89)90049-4 2809158

[44] Parkes J, Guha IN, Roderick P, Harris S, Cross R, Manos MM, et al Enhanced liver fibrosis (ELF) test accurately identifies liver fibrosis in patients with chronic hepatitis C. J Viral Hepat. 2011;18:23–31. 10.1111/j.1365-2893.2009.01263.x 20196799

[45] Nobili V, Parkes J, Bottazzo G, Marcellini M, Cross R, Newman D, et al Performance of ELF serum markers in predicting fibrosis stage in pediatric non-alcoholic fatty liver disease. Gastroenterology. 2009;136(1):160–7. 10.1053/j.gastro.2008.09.013 18992746

[46] Mayo MJ, Parkes J, Adams-huet B, Combes B, Mills AS, Markin RS, et al Prediction of clinical outcomes in primary biliary cirrhosis by serum enhanced liver fibrosis assay. Hepatology. 2008;48(5):1549–57. 10.1002/hep.22517 18846542 PMC2597274

[47] Xie Q, Zhou X, Huang P, Wei J, Wang W, Zheng S. The performance of enhanced liver fibrosis (ELF) test for the staging of liver fibrosis: a meta-analysis. PLoS One. 2014;9(4). 10.1371/journal.pone.0092772 PMC3988013 24736610

[48] Roy CC, Groleau V, Beaunoyer M, Marchand V. Clinical problem-solving: short bowel syndrome in an infant. Pediatr Child Heal. 2013;18(7):357–9. PMC3804635 24421709

[49] Savini S, Ascenzo RD, Biagetti C, Serpentini G, Pompilio A, Bartoli A, et al The effect of 5 intravenous lipid emulsions on plasma phytosterols in preterm infants receiving parenteral nutrition: a randomized clinical trial. Am J Clin Nutr. 2013;98:312–8. 10.3945/ajcn.112.056556 23761482

[50] Matsumoto M, Hada N, Sakamaki Y, Uno A, Shiga T, Tanaka C, et al An improved mouse model that rapidly develops fibrosis in non-alcoholic steatohepatitis. J Exp Pathol. 2013;94:93–103. 10.1111/iep.12008 PMC3607137 23305254

[51] Belkind-Gerson J, Carreon-Rodriguez A, Contreras-Ochoa CO, Estrada-Mondaca S, Parra-Carbrera MS. Fatty acids and neurodevelopment. J Pediatr Gastroenteroloy Nutr. 2008;47(4):7–9. 10.1097/MPG.0b013e3181818e3f 18667917

[52] Weiskirchen R, Tacke F. Relevance of autophagy in parenchymal and non-parenchymal liver cells for health and disease. Cells. 2019;8(16):1–13. 10.3390/cells8010016 PMC6357193 30609663

[53] Cober MP, Teitelbaum DH. Prevention of parenteral nutrition-associated liver disease: lipid minimization. Curr Opin Organ Transplant. 2010;15:330–3. 10.1097/MOT.0b013e328338c2da 20386446

[54] Nehra D, Fallon EM, Potemkin AK, Voss SD, Mitchell PD, Valim C, et al A comparison of two intravenous lipid emulsions: interim analysis of a randomized controlled trial. J Pediatr Enter Parenter Nutr. 2014;38(6):693–701. 10.1177/0148607113492549 PMC4635445 23770843

[55] Premji SS, Soraisham A, Chessell L, Sauve R. Dietary protien requirements for preterm infants in the neonatal period: past, present, and future In: Ling JR, editor. Dietary Protein: Research Trends. Nova Science; 2007 p. 63–99.

[56] Blanco CL, Hisey JC. Parenteral amino acid strategies for nutritional optimization in low birth weight infants In: Rajendram R, Patel VB, Preedy VR, editors. Diet and Nutrition in Critial Care. New York: Springer; 2015 p. 1957–69.

