# Supplementary figures and images for: Association of novel markers of liver disease with neonatal liver disease in premature baboons, Papio sp
Source: PLoS One. 2020 Mar 9;15(3):e0228985. doi: 10.1371/journal.pone.0228985 (PMC7062281; doi:10.1371/journal.pone.0228985)

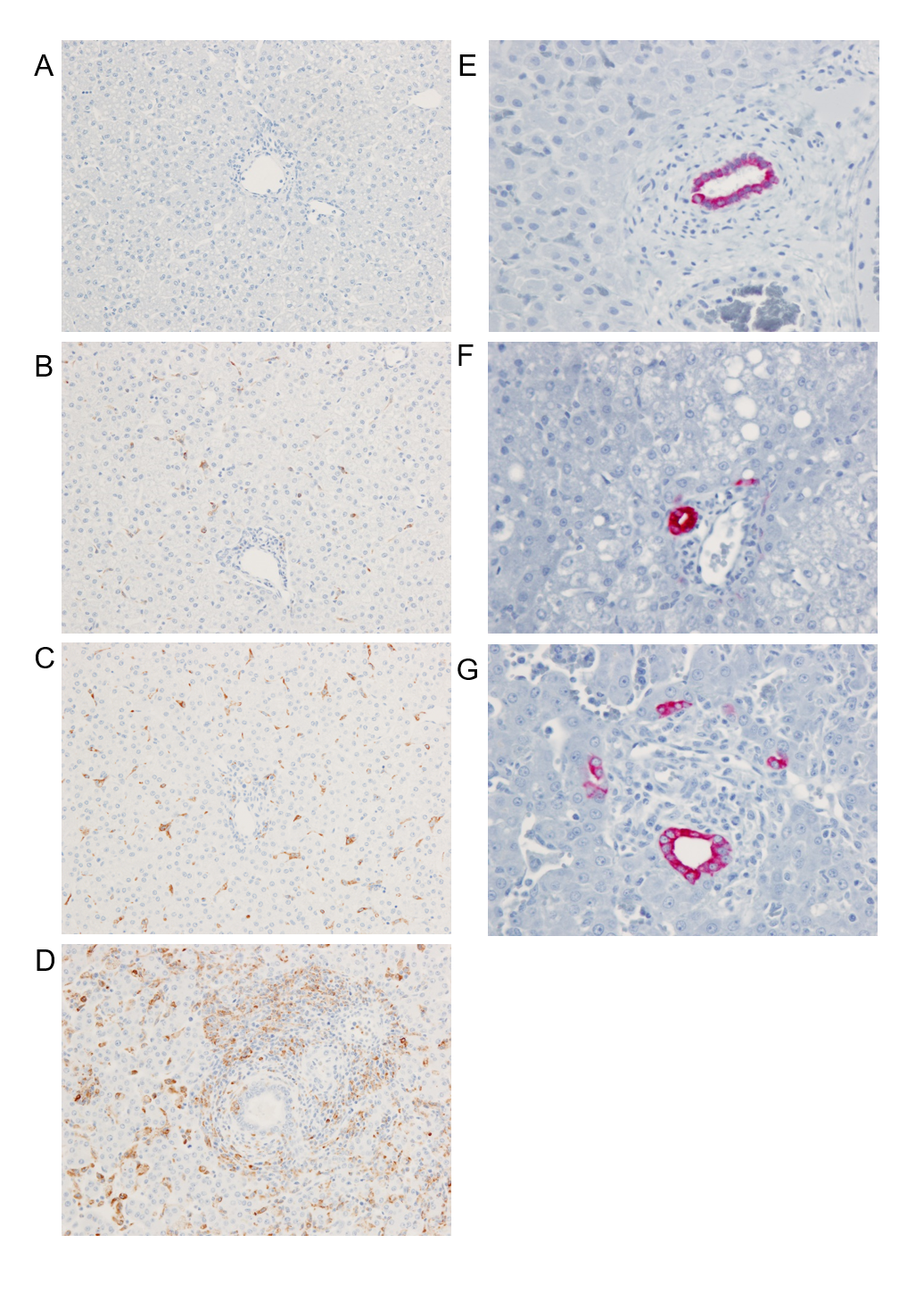

Supplement: S1 Fig — Representative images for each CD68 score and CK7 score are shown. Magnification is 20X for all images. [A] CD68 score of 0 is shown. No CD68+ macrophages are seen. [B] CD68 score of 1 is shown. Few scattered CD68+ macrophages are seen. [C] CD68 score of 2 is shown. A moderate number of CD68+ macrophages are seen. [D] CD68 score of 3 is shown. Marked infiltration of CD68+ macrophages is seen. [E] CK7 score of 0 is shown. No CK7+ progenitor cells are seen around this portal tract. [F] CK7 score of 1 is shown. Few single CK7+ progenitor cells are seen around this portal tract. [G] CK7 score of 2 is shown. Several clusters of CK7+ progenitor cells are seen around this portal tract. (TIF) [file pone.0228985.s001.tif]
